# Association Between Social Participation and Disability-free Life Expectancy in Japanese Older People: The Ohsaki Cohort 2006 Study

**DOI:** 10.2188/jea.JE20200574

**Published:** 2022-10-05

**Authors:** Sanae Matsuyama, Yoshitaka Murakami, Yukai Lu, Toshimasa Sone, Yumi Sugawara, Ichiro Tsuji

**Affiliations:** 1 Division of Epidemiology, Department of Health Informatics and Public Health, School of Public Health, Tohoku University Graduate School of Medicine, Sendai, Japan; 2 Department of Medical Statistics, Faculty of Medicine, Toho University, Tokyo, Japan

**Keywords:** social participation, community activity, disability-free life expectancy, life expectancy, prospective study

## Abstract

**Background:**

Although social participation has been reported to be associated with significantly lower risks of mortality and disability, to our knowledge, no study has estimated its impact on disability-free life expectancy (DFLE). Therefore, this study aimed to investigate the association between social participation and DFLE in community-dwelling older people.

**Methods:**

We analyzed 11-year follow-up data from a cohort study of 11,982 Japanese older adults (age ≥65 years) in 2006. We collected information on the number of social participations using a questionnaire. Using this information, we categorized the participants into four groups. DFLE was defined as the average number of years a person could expect to live without disability. The multistate life table method using a Markov model was employed for calculating DFLE.

**Results:**

The results revealed that DFLE according to the number of social participations was 17.8 years (95% confidence interval [CI], 17.3–18.2) for no activities, 20.9 (95% CI, 20.4–21.5) for one activity, 21.5 (95% CI, 20.9–22.0) for two activities, and 22.7 (95% CI, 22.1–23.2) for three activities in men, and 21.8 (95% CI, 21.5–22.2), 25.1 (95% CI, 24.6–25.6), 25.3 (95% CI, 24.7–25.9), and 26.7 years (95% CI, 26.1–27.4), respectively, in women. This difference in DFLE did not change after the participants were stratified for smoking, body mass index, physical activity, and depression.

**Conclusion:**

Social participation is associated with longer DFLE among Japanese older people; therefore, encouraging social participation at the population level could increase life-years lived in good health.

## INTRODUCTION

With the aging of the population, increasing attention is being paid to quality of life (QOL) rather than mere longevity. Healthy life expectancy (HLE), which is defined as the average number of years that a person can expect to live at a certain level of health, has recently been adopted to measure the state of health at the population level.^1^ Because HLE calculates both morbidity and mortality simultaneously, it can capture the both the quantity and quality of lived years.^2^^,^^3^

Extending HLE is regarded as the most important goal in national health promotion strategies, such as Healthy People 2030 in the United States^4^ and Health Japan 21 in Japan,^5^ as well as those conducted by the World Health Organization.^6^ Therefore, factors related to HLE are being studied around the world.

Previous studies have demonstrated that HLE is significantly longer among individuals with healthy lifestyle characteristics, such as nonsmoking status, normal weight, moderate physical activity levels, and healthy dietary habits.^7^^–^^12^ For instance, individuals with no risk factors (eg, current smoker, obesity, low physical inactivity levels) can be expected to live, on average, 8 years longer in good health and 6 years longer free of chronic disease compared with individuals with two or more risk factors.^9^

Social participation may be another factor in extending HLE because it has been shown to be associated with a significantly lower risk of mortality^13^^–^^16^ and disability.^14^^,^^17^^–^^19^ A previous study reported that individuals who seldom (only a few times a year) or never participate in social activities have a 25% higher risk of all-cause mortality than those who participate in social activities frequently.^15^ Our previous study reported that those participating in multiple social activities have a 30% lower risk of disability than those who do not.^17^ Additionally, social participation is a notable factor because various studies have examined the relationship between social participation and outcomes, such as depressive symptoms,^20^ dementia,^21^ and long-term care costs.^22^ However, to our knowledge, the association between social participation and HLE has not been investigated.

Therefore, this study aimed to investigate the association between social participation and HLE using data from a community-based prospective cohort study among Japanese older people. Among the various definitions of HLE,^1^ here, we focus on disability-free life expectancy (DFLE), which is defined as the average number of years that a person can expect to live without disability, because data on the incidence of disability are available from long-term care insurance (LTCI) information. To our knowledge, this is the first study to investigate to what extent social participation can extend DFLE. The results could be expected to provide insight into possible new strategies for extending DFLE and enhancing QOL among older people.

## METHODS

### Study cohort

The design of the Ohsaki Cohort 2006 Study has been described in detail elsewhere.^23^ In brief, the source population for the baseline survey comprised all older residents of Ohsaki City, Miyagi Prefecture, northeastern Japan, as of December 1, 2006 (ie, 31,694 men and women aged ≥65 years). The survey included question items about the frequency of participation in community activities, as well as those on body weight, height, smoking status, time spent walking per day, depression, and history of disease.

**Figure 1.**
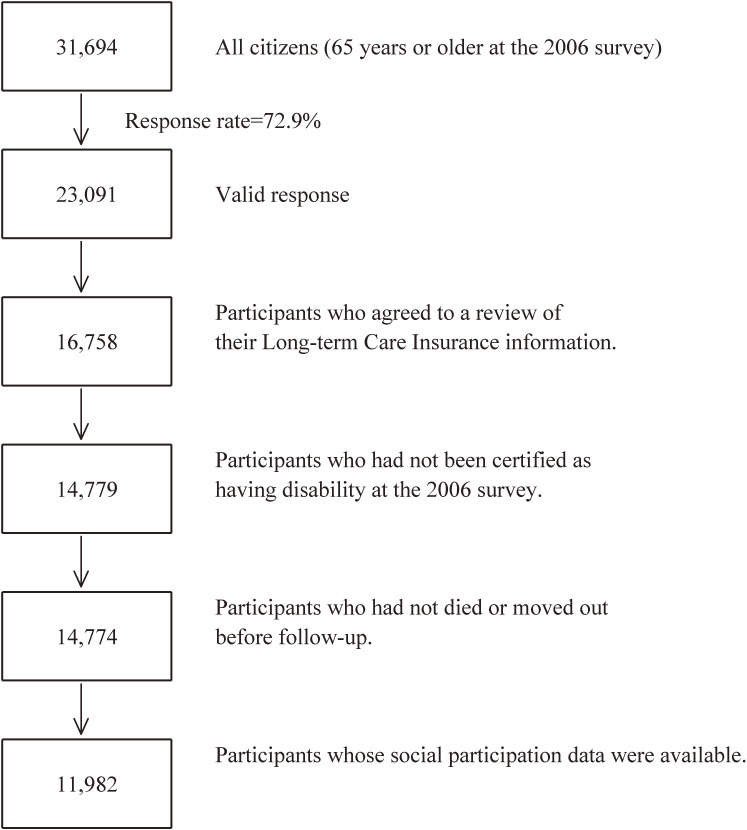
Flowchart of study participants

The baseline survey was conducted between December 1–15, 2006, and the follow-up survey was conducted between December 16, 2006, and November 30, 2017. The questionnaire was distributed by the heads of individual administrative districts and then collected by mail. For this analysis, 23,091 individuals who provided valid responses formed the study cohort. We excluded 6,333 individuals who had not provided written consent for a review of their LTCI information, 1,979 who had already been certified as having a disability by the LTCI (Support Level 1 or higher) before the start date of the follow-up survey, 5 who had died or moved before the start date of the follow-up survey, and 2,792 for whom information about social participation was unavailable. Therefore, 11,982 responses were finally analyzed for the purposes of this study (Figure 1).

### Exposure (social participation)

At the baseline, the participants were asked about their current social participation, which was classified into three categories: “Volunteering”, “Hobby activities”, and “Activities in neighborhood associations”. We asked the participants “How often do you participate in community activities?” and gave them some instruction about the types of such activities. Volunteering included community service activities, beautification activities, crime prevention, teaching children, providing childcare support, and so forth. Hobby activities included sports activities, karaoke, lifelong learning, and so forth. Activities in neighborhood associations included residents’ associations, senior clubs, associations for children and women, and so forth. In each category, the participants were also asked about their frequency of participation: “Do not participate”, “Less than once a month”, “1–3 times a month”, or “Once a week or more”. After we had collected the information on participation in community activities, we established the “number of social participations” as a parameter to indicate how many socialization categories (“Volunteering”, “Hobby activities”, and “Activities in neighborhood associations”) the individuals were participating in, and the activity was counted when the response was “Less than once a month” or more, and then the participants categorized as “None”, “One activity”, “Two activities”, or “Three activities” according to our previous study.^17^

### Outcomes

The study outcomes were incident disability according to national standards (LTCI Care Level 2 or higher: limited in performing activities of daily living [ADL]) and death.^24^ With these data, we calculated DFLE, which was defined as the average number of years that a person could expect to live without disability.

LTCI in Japan is a mandatory social insurance system that is meant to help frail older individuals carry out ADL. Everyone aged ≥40 years pays a premium, and everyone aged ≥65 years is eligible for formal caregiving services depending on the level (from Support Level 1 to Support Level 2, and from Care Level 1 to Care Level 5). LTCI certification was found to be associated with the ability to perform ADL in a community-based study,^25^ and has been used in epidemiologic studies as a measure of incident functional disability among older individuals.^26^^,^^27^ Data regarding incident functional disability, death, or emigration during follow-up were transferred from the Ohsaki City Government through an agreement about the secondary use of data. All data were transferred from the Ohsaki City Government yearly each December under the agreement on Epidemiologic Research and Privacy Protection.

### Ethical issues

We considered the return of a completed questionnaire to imply consent to participate in the study, including the baseline survey data and subsequent follow-up. We also confirmed information regarding LTCI certification status after obtaining written consent. The Ethics Committee of Tohoku University Graduate School of Medicine (Sendai, Japan) reviewed and approved the study protocol (approval code: 2006-206).

### The multistate life table (MSLT) method

The multistate life table (MSLT) method was employed to conduct the analyses. This method for analyzing HLE was first introduced by Laditka and Wolf.^28^ In our analysis, a Markov transitions model for disability and mortality consisted of the following three states; two non-absorbing states (non-disabled and disabled) and one absorbing state (dead). There were four possible health transitions over time: (a) from non-disabled to disabled (the incidence of a disabled status), (b) from disabled to non-disabled (recovery from a disabled status), (c) from non-disabled to dead, and (d) from disabled to dead. In our model, we allowed retention status for the non-disabled and disabled.

### Statistical analysis

**Figure 2.**
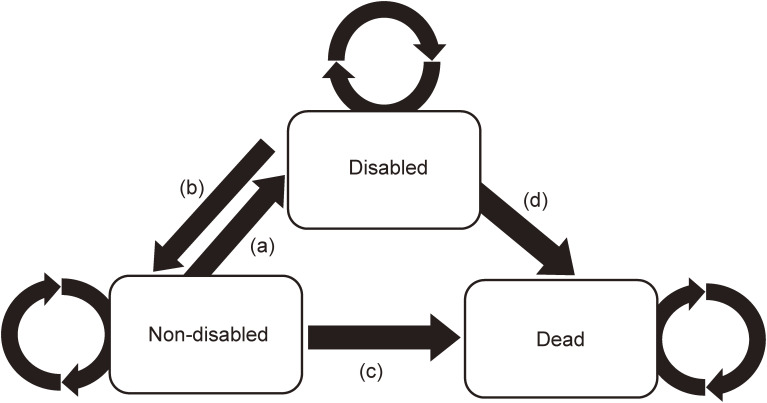
The Markov model (the multistate life table methods) applied in our study. In this scheme, three states (non-disabled, disabled, and dead) and four transitions over time were shown: that is (a) from non-disabled to disabled (an incidence of a disabled status), (b) from disabled to non-disabled (a recovery from a disabled status), (c) from non-disabled to dead, and (d) from disabled to dead. Each circulated arrows showed the retention process.

The DFLE in both non-disabled and disabled was computed using Interpolated Markov Chain (IMaCh) software (version 0.98r7), which was developed at the Institut national d’études démographiques by Brouard and Lièvre.^29^ This well-known software has been widely applied in several recent studies to compute HLE.^30^^–^^32^ The program has been described in detail in a previous paper,^33^ so we only provide a brief description here. In our analysis, a Markov model (shown in Figure 2) was created to calculate DFLE. Four transition probabilities of the Markov model, which was age- and group-specific, were estimated using multinomial logistic regression. We implemented these probabilities in the MSLT and calculated the total life expectancy (TLE), DFLE, and duration with disability for each subgroup. We calculated DFLE of each component of social participation (Volunteering, Hobby activities, and Activities in neighborhood associations) using IMaCh. We categorized the participants into four groups according to the number of the social participations. We then calculated the group-specific DFLEs using IMaCh.

Because the IMaCh program cannot adjust for confounding factors, we performed five stratified analyses by smoking status (never or former, or current), body mass index (BMI) (two categories: BMI 18.5 to <25 kg/m^2^ or BMI <18.5 or ≥25.0 kg/m^2^), time spent walking (≥0.5 hours/day or <0.5 hours/day), depression (free of depression or depression), and the number of non-communicable disease risks (0, 1, or 2 or 3). We defined non-communicable disease risk as follows: 1) current smoker, 2) BMI <18.5 or BMI ≥25.0, and 3) time spent walking <0.5 hours/day. We then established the “number of non-communicable disease risks” as a parameter to indicate how many of the non-communicable disease risk the participants had, and stratified the responses as “no risk factor”, “one risk factor”, or “two or three risk factors”.

Depression was measured in the baseline survey using the Depression and Suicide Screen (DSS), which was developed by Fujisawa et al in Japanese as a brief screening instrument for depression and suicidal ideation in aged persons.^34^ The DSS is composed of the following five items: 1) “Is your life pretty full?”, 2) “Do you still enjoy doing the things you used to do?”, 3) “Do you think it is too much trouble to do the things you used to do?”, 4) “Do you feel that you are a useful person who is needed by others?”, and 5) “Do you feel tired without any specific reason?”. For items 1, 2, and 4, responses of “yes” are scored 1 and responses of “no” are scored 0; for items 3 and 5, responses of “yes” are scored 0 and responses of “no” are scored 1. The cutoff of 1 (≤1 vs >1) produced satisfactory sensitivity and specificity for detecting depression (70.5% and 72.9%, respectively),^34^ so we defined DSS scores ≥2 as “depression”.

The MSLT method was performed using the IMaCh software program, and the data preparation and description were performed by using SAS version 9.4 (SAS Inc., Cary, NC, USA).

## RESULTS

### Participants’ characteristics

Among the 11,982 participants, the proportion of men was 46.0%, the mean age was 73.6 (standard deviation, 6.0) years, and only 369 individuals were lost to follow-up because of emigration from the study area; thus, the follow-up rate was 96.9%.

Table 1 shows the characteristics of the study participants according to the number of social participations. The mean age was lower in the social participation group, and those with frequent social participation included higher proportions of males and current smokers and lower proportions of those with depression and those walking less than 0.5 hour per day.

**Table 1.** Baseline characteristics of the study participants according to the number of social participation (*n* = 11,982)

	Number of social participation				*P*-values^a^

	None	One activity	Two activities	Three activities	
Number of subjects	4,641	2,630	2,084	2,627	
Age, years, mean (SD)	75.1 (6.5)	73.1 (5.7)	72.7 (5.5)	72.1 (4.9)	<0.001
Men, %	39.0	42.0	50.9	58.4	<0.001
Body mass index, kg/m^2^, mean (SD)	23.4 (3.7)	23.5 (3.3)	23.6 (3.1)	23.9 (3.0)	<0.001
Current smokers, %	13.1	13.1	13.6	14.2	<0.001
Time spent walking <0.5 h/d, %	46.1	33.7	30.7	24.5	<0.001
Depression,^b^ %	40.2	26.0	20.4	14.0	<0.001
History of disease, %					
Hypertension	45.0	44.9	41.9	41.1	0.002
Diabetes mellitus	12.6	11.9	11.0	12.0	0.334
Stroke	3.8	2.4	2.1	2.0	<0.001
Myocardial infarction	5.6	4.6	5.5	4.0	0.011
Cancer	9.7	8.6	9.1	8.6	0.278

SD, standard deviation.

^a^Obtained by using *X*^2^ test for variables of proportion and one-factor ANOVA for continuous variables (missing value exclude).

^b^Depression and Suicide Screen score ≥2.

Table 2 shows the distribution of the outcomes in 2017 among the participants by number of social participations and sex. For men, 2,938 (53.3%) were non-disabled, 336 (6.1%) were disabled, and 2,091 (38.0%) were dead. For women, 4,061 (62.7%) were non-disabled, 751 (11.6%) were disabled, and 1,435 (22.2%) were dead.

**Table 2.** The distribution of participants in the outcome in 2017 by the number of social participation (*n* = 11,982)

Outcome	Number of social participation									

	None		One activity		Two activities		Three activities		Total	
**Men**										
Non-disabled, %	702	(12.7)	615	(11.2)	622	(11.3)	1,000	(18.1)	2,938	(53.3)
Disabled, %	125	(2.3)	68	(1.2)	63	(1.1)	80	(1.5)	336	(6.1)
Dead, %	933	(16.9)	393	(7.2)	344	(6.3)	421	(7.6)	2,091	(38.0)
Emigrated, %	48	(0.9)	30	(0.5)	32	(0.6)	32	(0.6)	142	(2.6)
Total, %	1,808	(32.8)	1,106	(20.0)	1,061	(19.3)	1,533	(27.8)	5,508	(100.0)
**Women**										
Non-disabled, %	1,444	(22.3)	1,034	(16.0)	737	(11.3)	846	(13.1)	4,061	(62.7)
Disabled, %	394	(6.1)	177	(2.7)	101	(1.6)	79	(1.2)	751	(11.6)
Dead, %	884	(13.7)	264	(4.0)	159	(2.5)	128	(2.0)	1,435	(22.2)
Emigrated, %	111	(1.7)	49	(0.8)	26	(0.4)	41	(0.6)	227	(3.5)
Total, %	2,833	(43.8)	1,524	(23.5)	1,023	(15.8)	1,094	(16.9)	6,474	(100.0)

### Association between social participation and disability-free life expectancy (DFLE)

Table 3 shows DFLE, duration with disability, and TLE by the number of social participations for men and women at age 65 years. The number of social participations was associated with longer DFLE and TLE for both sexes. DFLE was 17.8 years (95% confidence interval [CI], 17.3–18.2) for no activity, 20.9 (95% CI, 20.4–21.5) for one, 21.5 (95% CI, 20.9–22.0) for two, and 22.7 (95% CI, 22.1–23.2) for three in men, and 21.8 (95% CI, 21.5–22.2), 25.1 (95% CI, 24.6–25.6), 25.3 (95% CI, 24.7–25.9), and 26.7 years (95% CI, 26.1–27.4), respectively, in women. The difference in DFLE between the no activities group and the three activities group was about 5 years for both sexes (17.8 vs 22.7 years for men and 21.8 vs 26.7 years for women).

**Table 3.** DFLE, duration with disability and TLE at 65 years by the number of social participation (*n* = 11,982)

Number of social participation	Number of participants	DFLE	(95% CI)	Duration with disability	(95% CI)	TLE	(95% CI)
**Men**							
None	1,808	17.8	(17.3–18.2)	0.9	(0.8–1.0)	18.7	(18.2–19.1)
One activity	1,106	20.9	(20.4–21.5)	1.0	(0.9–1.0)	21.9	(21.3–22.5)
Two activities	1,061	21.5	(20.9–22.0)	1.0	(0.9–1.1)	22.5	(21.8–23.1)
Three activities	1,533	22.7	(22.1–23.2)	1.0	(0.9–1.1)	23.7	(23.1–24.3)
**Women**							
None	2,833	21.8	(21.5–22.2)	3.9	(3.5–4.2)	25.7	(25.2–26.2)
One activity	1,524	25.1	(24.6–25.6)	4.7	(3.9–5.5)	29.8	(28.9–30.7)
Two activities	1,023	25.3	(24.7–25.9)	4.2	(3.4–4.9)	29.5	(28.5–30.4)
Three activities	1,094	26.7	(26.1–27.4)	4.0	(3.1–4.8)	30.7	(29.6–31.7)

CI, confidence interval; DFLE, disability-free life expectancy; TLE, total life expectancy.

Table 4 shows DFLE, duration with disability, and TLE by the frequency of participation in each activity for men and women at age 65 years. Overall, in all groups and for both sexes, those engaging in social participation, even if less than once a month (ie, several times a year), could expect a longer DFLE than those engaging in no activities. The difference in DFLE between the no activities group and the once a week or more group was about 3.5 years in both sexes (men: 19.2 vs 23.0 years for volunteering, 18.8 vs 22.5 for hobby activities, and 18.8 vs 22.3 for activities in neighborhood associations; women: 23.1 vs 26.5, 22.5 vs 26.0, and 22.7 vs 25.9 years, respectively).

**Table 4.** DFLE, duration with disability, and TLE at 65 years by frequency of each social participation (*n* = 11,982)

Frequency of each social participation	DFLE	(95% CI)	Duration with disability	(95% CI)	TLE	(95% CI)
**Men**						
**Volunteering**						
None	19.2	(18.8–19.5)	0.9	(0.9–1.0)	20.1	(19.8–20.5)
<1 time/month	22.1	(21.4–22.7)	0.9	(0.9–1.1)	23.0	(22.4–23.7)
1–3 time/month	22.5	(21.7–23.3)	1.0	(0.9–1.2)	23.5	(22.7–24.4)
≥1 time/week	23.0	(21.9–24.0)	1.0	(0.9–1.2)	24.0	(22.9–25.1)
**Hobby activities**						
None	18.8	(18.4–19.2)	0.9	(0.9–1.0)	19.7	(19.3–20.1)
<1 time/month	21.6	(20.9–22.3)	1.0	(0.9–1.1)	22.6	(21.8–23.3)
1–3 time/month	22.3	(21.6–22.9)	1.0	(0.9–1.1)	23.3	(22.6–23.9)
≥1 time/week	22.5	(21.8–23.1)	0.9	(0.8–1.1)	23.4	(22.7–24.1)
**Activities in neighborhood association**						
None	18.8	(18.4–19.2)	0.9	(0.8–1.0)	19.7	(19.3–20.1)
<1 time/month	21.4	(20.8–21.9)	1.0	(0.9–1.1)	22.4	(21.8–23.0)
1–3 time/month	22.2	(21.6–22.8)	1.0	(0.9–1.1)	23.2	(22.6–23.8)
≥1 time/week	22.3	(21.4–23.2)	1.1	(1.0–1.3)	23.4	(22.5–24.4)
**Women**						
**Volunteering**						
None	23.1	(22.8–23.4)	4.1	(3.7–4.4)	27.2	(26.7–27.6)
<1 time/month	26.0	(25.3–26.7)	3.9	(3.0–4.8)	29.9	(28.8–31.0)
1–3 time/month	26.3	(25.5–27.2)	4.5	(3.1–5.9)	30.8	(29.3–32.4)
≥1 time/week	26.5	(25.4–27.7)	4.5	(2.5–6.4)	31.0	(28.8–33.1)
**Hobby activities**						
None	22.5	(22.2–22.8)	4.0	(3.7–4.3)	26.5	(26.1–27.0)
<1 time/month	25.3	(24.6–26.1)	3.4	(2.6–4.1)	28.7	(27.6–29.7)
1–3 time/month	26.1	(25.5–26.7)	4.5	(3.6–5.5)	30.6	(29.5–31.7)
≥1 time/week	26.0	(25.4–26.7)	4.4	(3.4–5.3)	30.4	(29.2–31.5)
**Activities in neighborhood association**						
None	22.7	(22.3–23.0)	3.9	(3.6–4.2)	26.6	(26.1–27.1)
<1 time/month	24.9	(24.4–25.5)	4.5	(3.7–5.2)	29.4	(28.4–30.3)
1–3 time/month	26.0	(25.4–26.6)	4.2	(3.4–5.0)	30.2	(29.2–31.2)
≥1 time/week	25.9	(24.9–26.9)	5.3	(3.3–7.3)	31.2	(29.2–33.3)

CI, confidence interval; DFLE, disability-free life expectancy; TLE, total life expectancy.

When we compared each activity, the DFLE of volunteering was slightly longer at all frequencies. DFLE in the once a week or more category was 23.0 years (95% CI, 21.9–24.0) for volunteering, 22.5 (95% CI, 21.8–23.1) for hobby activities, and 22.3 (95% CI, 21.4–23.2) for neighborhood associations in men, and 26.5 (95% CI, 25.4–27.7), 26.0 (95% CI, 25.4–26.7), and 25.9 years (95% CI, 24.9–26.9), respectively, in women.

### Stratified analysis

As indicated in Table 1, the number of social participations was associated with smoking status, BMI, time spent walking, and depression. Because the IMaCh program cannot adjust for confounding factors, we performed several stratified analyses. In a stratified analysis by smoking status, the association between the number of social participations and DFLE did not differ between never/former smokers and current smokers. In other words, the difference in DFLE between the no activities group and the three activities group was about 5 years for both smoking statuses (eTable 1). We also observed a consistent association when the participants were stratified by BMI (eTable 2), time spent walking (eTable 3), or depression (eTable 4). Additionally, a difference in DFLE between the no activities group and the three activities group was consistently observed, even when we stratified the participants by the number of non-communicable disease risks, which combined smoking status, BMI, and time spent walking (eTable 5). From the above, the association between social participation and DFLE was independent of confounding factors such as smoking status, BMI, time spent walking, and depression.

## DISCUSSION

In this cohort study, we estimated DFLE according to the number of social participations based on data from 11 years of prospective observations of 11,982 older individuals in Japan. We found that the number of social participations was associated with longer DFLE. A 5-year difference in DFLE was observed between those who participated in no activities and those who participated in three activities for both men and women. The impact of social participation on DFLE was consistent even after we stratified the participants by smoking status, BMI, time spent walking, and the number of non-communicable disease risks. On the other hand, the absolute value of duration with disability was constant over the categories of social participation. To our knowledge, this is the first study to estimate the impact of social participation on DFLE.

The DFLE previously reported by the Ministry of Health, Labour and Welfare of Japan was 79.5 years for men and 83.8 years for women, and the definition of DFLE was the same as that used in this study (ie, LTCI Care Level 2 or higher).^24^ In this study, DFLE at age 65 years was calculated, so when 65 years was added to DFLE in Table 3, DFLE was 82.8 years for men and 86.8 years for women in the no activities group. The DFLE in this study was about 3 years higher than national estimates. This difference might be attributable to the fact that the baseline survey sample comprised only community-dwelling people and excluded institutionalized patients and participants who had already been certified as having a disability by LTCI before the follow-up survey.

Previous studies have reported that social participation is associated with lower risks of mortality^13^^–^^16^ and disability,^14^^,^^17^^–^^19^ and the mechanisms of these associations were described as maintaining cognitive and physical functions through social relationships and roles, developing social networks, accessing material resources or various forms of social support, improving health literacy, and obtaining a feeling of comfort and joy in pursuing preferred activities. These benefits from social participation would lead to not only longevity, but also healthy aging.

We consider the impact of the 5-year difference in DFLE between the no activities group and the three activities group. Because no previous study has examined the relationship between social participation and HLE, we carry out a comparison with studies that have already clarified the relationship between non-communicable disease risks and HLE. According to four cohort studies in Europe, the difference in disease-free life expectancy between never or former versus current smokers was 2.0–5.1 years for men and 1.8–4.9 years for women, regular physical activity versus inactivity was 2.7–7.2 years for men and 2.8–6.7 years for women, and BMI <30 versus obesity was 2.9–5.7 years for men and 3.0–5.6 years for women.^9^^,^^11^ In the present study, we observed a consistent difference in DFLE due to social participation, which was about 5 years for both sexes. The impact of social participation on DFLE was compatible with that of non-communicable disease risks.

When we investigated the association between the frequency of participation in each activity and DFLE, the difference in DFLE between the no activities group and once a week or more group was about 3.5 years for both sexes; the impact was smaller than that for the number of social participations. Therefore, to extend HLE, it may be important to participate in various types of activities. A previous study reported that the odds ratios of mortality and disability were lower as the number of social participations increased.^35^ Participation in various types of activities, such as volunteering, hobby activities, and activities in neighborhood association, is expected to expand the community in which residents belong, increase the number of people they will meet, and provide substantial physical and cognitive stimulation.^36^^,^^37^

This study had several strengths. First, we applied the MSLT method to calculate DFLE. Although several approaches, such as the Cox model, exist for calculating DFLE, the MSLT method has the advantage of being able to deal with the recovery process (the transition from disabled to non-disabled) using a Markov model. Second, this was a large population-based cohort study involving 11,982 persons. Third, the response rate was relatively high (72.9%). Fourth, the follow-up period was sufficiently long to estimate DFLE in older people. Finally, few participants were lost during follow-up (3.1%).

However, this study also had several limitations. First, because not all candidates had applied for LTCI certification, this study may not have been completely free from detection bias. Second, 6,333 participants who did not agree to have their LTCI information reviewed were excluded from the analyses, and we have compared their characteristics with those of the participants who agreed (eTable 6). As a result, those who disagreed tended to be women, current smokers, have depression, and be free from hypertension, stroke, myocardial infarction, or cancer. Third, the questionnaire on social participation had not been evaluated for reliability and validity. Fourth, because the IMaCh program cannot adjust for confounding factors, DFLE could have been overestimated; however, our results were consistent even when we stratified the participants by potential confounding factors such as smoking status, BMI, physical activity, and depression.

In conclusion, the results of this study suggest the substantial impact of social participation on longer DFLE among older people. A 5-year difference in DFLE was observed between those who participated in no activities compared with those who participated in all three activities; this association was consistent among both men and women. These findings suggest that encouraging social participation at the population level could increase life-years lived in good health among community-dwelling older people.

## SUPPLEMENTARY MATERIAL

eTable 1. DFLE, duration with disability, and TLE at 65 years according to the number of social participation by smoking status

eTable 2. DFLE, duration with disability, and TLE at 65 years according to the number of social participation by BMI

eTable 3. DFLE, duration with disability, and TLE at 65 years according to the number of social participation by time spent walking

eTable 4. DFLE, duration with disability, and TLE at 65 years according to the number of social participation by depression

eTable 5. DFLE, duration with disability, and TLE at 65 years according to the number of social participation by the number of non-communicable disease risks

eTable 6. Baseline characteristics according to whether or not agreed to a review of their LTCI information
