# Supplementary material for: Association Between Social Participation and Disability-free Life Expectancy in Japanese Older People: The Ohsaki Cohort 2006 Study
Source: J Epidemiol. 2022 Oct 5;32(10):456–63. doi: 10.2188/jea.JE20200574 (PMC9424187; doi:10.2188/jea.JE20200574)
Supplement: Supplementary file 1 [file je-32-456-s001.pdf]

eTable 1. DFLE, duration with disability, and TLE at 65 years according to the number of social participation by smoking status

Number of social participation	DFLE	(95% CI)	Duration with disability	(95% CI)	TLE	(95% CI)
**Men**						
**Never or former smokers**						
None	18.5	(18.1–19.0)	1.0	(0.9–1.0)	19.5	(19.0–20.0)
One activity	21.7	(21.2–22.3)	1.0	(0.9–1.1)	22.7	(22.1–23.3)
Two activities	22.2	(21.6–22.8)	1.1	(1.0–1.2)	23.3	(22.6–23.9)
Three activities	23.4	(22.8–24.0)	1.1	(0.9–1.2)	24.5	(23.9–25.1)
**Current smokers**						
None	15.8	(15.2–16.4)	0.7	(0.7–0.9)	16.5	(15.9–17.2)
One activity	18.8	(18.1–19.5)	0.8	(0.7–0.9)	19.6	(18.8–20.3)
Two activities	19.3	(18.5–20.1)	0.9	(0.8–1.0)	20.2	(19.4–21.0)
Three activities	20.5	(19.7–21.2)	0.8	(0.7–1.0)	21.3	(20.5–22.1)
**Women**						
**Never or former smokers**						
None	21.8	(21.4–22.2)	3.8	(3.5–4.2)	25.6	(25.1–26.2)
One activity	25.0	(24.5–25.6)	4.7	(3.8–5.6)	29.7	(28.7–30.7)
Two activities	25.3	(24.6–25.9)	4.3	(3.4–5.3)	29.6	(28.5–30.7)
Three activities	26.7	(26.0–27.4)	4.0	(3.0–4.9)	30.7	(29.6–31.8)
**Current smokers**						
None	19.3	(18.6–20.0)	3.0	(2.1–4.0)	22.3	(21.2–23.4)
One activity	22.4	(21.6–23.3)	3.7	(2.3–5.1)	26.1	(24.6–27.7)
Two activities	22.7	(21.8–23.6)	3.5	(2.2–4.8)	26.2	(24.7–27.7)
Three activities	24.1	(23.2–25.0)	3.2	(2.0–4.4)	27.3	(25.9–28.7)

CI, confidence interval; DFLE, disability-free life expectancy; TLE, total life expectancy.

eTable 2. DFLE, duration with disability, and TLE at 65 years according to the number of social participation by BMI

Number of social participation	DFLE	(95% CI)	Duration with disability	(95% CI)	TLE	(95% CI)
**Men**						
**18.5≤ BMI <25.0**						
None	17.8	(17.3–18.3)	0.9	(0.8–1.0)	18.7	(18.2–19.3)
One activity	20.9	(20.3–21.5)	1.0	(0.8–1.0)	21.9	(21.2–22.5)
Two activities	21.5	(20.9–22.2)	1.1	(0.9–1.1)	22.6	(21.9–23.3)
Three activities	22.9	(22.3–23.6)	1.1	(0.9–1.2)	24.0	(23.3–24.6)
**BMI<18.5 or 25.0≤ BMI**						
None	17.8	(17.3–18.4)	0.9	(0.8–1.0)	18.7	(18.1–19.3)
One activity	20.9	(20.2–21.6)	0.9	(0.8–1.0)	21.8	(21.1–22.6)
Two activities	21.6	(20.8–22.3)	1.0	(0.9–1.1)	22.6	(21.8–23.3)
Three activities	23.0	(22.3–23.6)	1.0	(0.9–1.1)	24.0	(23.3–24.7)
**Women**						
**18.5≤ BMI <25.0**						
None	22.0	(21.6–22.4)	3.8	(3.4–4.2)	25.8	(25.2–26.4)
One activity	25.1	(24.5–25.7)	4.7	(3.8–5.6)	29.8	(28.8–30.9)
Two activities	25.4	(24.8–26.1)	4.6	(3.5–5.5)	30.0	(28.8–31.1)
Three activities	27.0	(26.2–27.7)	4.0	(3.1–5.0)	31.0	(29.8–32.2)
**BMI<18.5 or 25.0≤ BMI**						
None	22.2	(21.7–22.7)	3.7	(3.2–4.2)	25.9	(25.2–26.6)
One activity	25.3	(24.6–25.9)	4.6	(3.6–5.6)	29.9	(28.7–31.0)
Two activities	25.6	(24.9–26.4)	4.4	(3.4–5.5)	30.0	(28.8–31.3)
Three activities	27.1	(26.4–27.9)	4.0	(3.0–5.0)	31.1	(29.9–32.3)

BMI, body mass index; CI, confidence interval; DFLE, disability-free life expectancy; TLE, total life expectancy.

eTable 3. DFLE, duration with disability, and TLE at 65 years according to the number of social participation by time spent walking

Number of social participation	DFLE	(95% CI)	Duration with disability	(95% CI)	TLE	(95% CI)
**Men**						
**≥0.5 hours/day**						
None	18.6	(18.2–19.1)	1.5	(1.3–1.6)	20.1	(19.5–20.6)
One activity	21.6	(21.0–22.2)	1.5	(1.3–1.8)	23.1	(22.5–23.8)
Two activities	22.1	(21.5–22.7)	1.7	(1.4–1.9)	23.8	(23.1–24.5)
Three activities	23.2	(22.6–23.8)	1.7	(1.4–1.9)	24.9	(24.2–25.5)
**<0.5 hours/day**						
None	16.4	(15.9–16.9)	1.4	(1.3–1.6)	17.8	(17.3–18.4)
One activity	19.3	(18.6–19.9)	1.5	(1.3–1.8)	20.8	(20.1–21.5)
Two activities	19.8	(19.1–20.5)	1.6	(1.4–1.9)	21.4	(20.7–22.2)
Three activities	20.8	(20.2–21.5)	1.7	(1.4–1.9)	22.5	(21.7–23.2)
**Women**						
**≥0.5 hours/day**						
None	23.0	(22.6–23.5)	4.0	(3.5–4.3)	27.0	(26.3–27.6)
One activity	26.1	(25.5–26.7)	4.3	(3.7–5.0)	30.4	(29.5–31.3)
Two activities	26.3	(25.6–26.9)	4.3	(3.6–5.1)	30.6	(29.6–31.6)
Three activities	27.5	(26.8–28.2)	4.4	(3.6–5.2)	31.9	(30.8–32.9)
**<0.5 hours/day**						
None	20.8	(20.4–21.3)	4.0	(3.6–4.3)	24.8	(24.2–25.3)
One activity	23.8	(23.2–24.4)	4.4	(3.7–5.0)	28.2	(27.3–29.1)
Two activities	24.0	(23.3–24.7)	4.4	(3.7–5.2)	28.4	(27.4–29.4)
Three activities	25.2	(24.5–25.9)	4.4	(3.6–5.3)	29.6	(28.6–30.7)

CI, confidence interval; DFLE, disability-free life expectancy; TLE, total life expectancy.

eTable 4. DFLE, duration with disability, and TLE at 65 years according to the number of social participation by depression

Number of social participation	DFLE	(95% CI)	Duration with disability	(95% CI)	TLE	(95% CI)
**Men**						
**Free of depression**						
None	18.7	(18.2–19.2)	0.9	(0.8–1.0)	19.6	(19.1–20.1)
One activity	21.8	(21.2–22.3)	0.9	(0.8–1.0)	22.7	(22.1–23.3)
Two activities	22.2	(21.6–22.8)	1.0	(0.9–1.1)	23.2	(22.5–23.9)
Three activities	23.2	(22.6–23.8)	1.0	(0.9–1.1)	24.2	(23.6–24.8)
**Depression**						
None	15.9	(15.4–16.4)	0.9	(0.8–1.0)	16.8	(16.3–17.4)
One activity	18.8	(18.2–19.5)	1.0	(0.9–1.1)	19.8	(19.1–20.5)
Two activities	19.2	(18.5–19.9)	1.0	(0.9–1.1)	20.2	(19.5–21.0)
Three activities	20.2	(19.5–20.9)	1.0	(0.9–1.2)	21.2	(20.5–21.9)
**Women**						
**Free of depression**						
None	23.2	(22.8–23.6)	3.7	(3.3–4.1)	26.9	(26.3–27.5)
One activity	26.2	(25.6–26.8)	4.5	(3.7–5.4)	30.7	(29.8–31.8)
Two activities	26.5	(25.8–27.1)	4.1	(3.3–4.9)	30.6	(29.5–31.6)
Three activities	27.5	(26.8–28.2)	4.0	(3.1–4.9)	31.5	(30.4–32.6)
**Depression**						
None	20.1	(19.6–20.5)	4.0	(3.6–4.4)	24.1	(23.5–24.7)
One activity	23.0	(22.4–23.6)	5.0	(4.1–5.9)	28.0	(26.9–29.1)
Two activities	23.2	(22.5–23.9)	4.5	(3.6–5.5)	27.7	(26.6–28.9)
Three activities	24.2	(23.5–25.0)	4.4	(3.3–5.4)	28.6	(27.4–29.8)

CI, confidence interval; DFLE, disability-free life expectancy; TLE, total life expectancy.

eTable 5. DFLE, duration with disability, and TLE at 65 years according to the number of social participation by the number of non-communicable disease risks

Number of social participation	DFLE	(95% CI)	Duration with disability	(95% CI)	TLE	(95% CI)
**Men**						
**0 risk factor**						
None	19.5	(18.9–20.1)	1.1	(0.9–1.1)	20.6	(19.9–21.2)
One activity	22.5	(21.8–23.2)	1.0	(0.9–1.1)	23.5	(22.8–24.3)
Two activities	23.0	(22.3–23.7)	1.1	(1.0–1.2)	24.1	(23.3–24.9)
Three activities	24.4	(23.7–25.1)	1.1	(1.0–1.3)	25.5	(24.8–26.3)
**1 risk factor**						
None	17.6	(17.0–18.1)	0.8	(0.8–1.0)	18.4	(17.9–19.0)
One activity	20.4	(19.8–21.1)	0.9	(0.8–1.0)	21.3	(20.6–22.0)
Two activities	21.0	(20.3–21.7)	1.0	(0.9–1.1)	22.0	(21.2–22.7)
Three activities	22.4	(21.7–23.0)	0.9	(0.9–1.1)	23.3	(22.6–24.1)
**2 or 3 risk factors**						
None	16.6	(16.0–17.3)	0.9	(0.7–0.9)	17.5	(16.8–18.2)
One activity	19.5	(18.7–20.2)	0.8	(0.7–0.9)	20.3	(19.5–21.1)
Two activities	20.0	(19.2–20.8)	1.0	(0.8–1.1)	21.0	(20.1–21.8)
Three activities	21.4	(20.6–22.2)	0.9	(0.8–1.1)	22.3	(21.5–23.2)
**Women**						
**0 risk factor**						
None	23.3	(22.8–23.9)	3.8	(3.3–4.4)	27.1	(26.3–27.9)
One activity	26.3	(25.5–27.0)	4.7	(3.6–5.8)	31.0	(29.7–32.2)
Two activities	26.5	(25.7–27.3)	4.8	(3.6–6.1)	31.3	(29.9–32.8)
Three activities	28.0	(27.2–28.8)	4.1	(3.1–5.2)	32.1	(30.8–33.4)

**1 risk factor**						
None	21.7	(21.2–22.2)	3.7	(3.2–4.1)	25.4	(24.7–26.1)
One activity	24.6	(23.9–25.2)	4.5	(3.6–5.6)	29.1	(28.0–30.3)
Two activities	24.9	(24.1–25.6)	4.7	(3.5–5.9)	29.6	(28.2–31.0)
Three activities	26.4	(25.6–27.1)	4.0	(3.0–5.2)	30.4	(29.1–31.7)
**2 or 3 risk factors**						
None	20.9	(20.2–21.5)	3.4	(2.9–4.0)	24.3	(23.5–25.2)
One activity	23.7	(23.0–24.5)	4.3	(3.2–5.3)	28.0	(26.7–29.3)
Two activities	24.0	(23.2–24.9)	4.5	(3.2–5.7)	28.5	(27.0–30.0)
Three activities	25.5	(24.6–26.4)	3.8	(2.7–4.9)	29.3	(27.9–30.7)

CI, confidence interval; DFLE, disability-free life expectancy; TLE, total life expectancy.

eTable 6. Baseline characteristics according to whether or not agreed to a review of their LTCI information

	Participants who disagree to a review to their LTCI information	Participants who agree to a review to their LTCI information	*P*-values[a]
Number of subjects	6333	16758	
Age, years, mean (SD)	74.9 (6.5)	74.9 (6.6)	0.756
Men, %	37.1	43.3	<0.001
Body mass index, kg/m^2, mean (SD)	23.5 (3.5)	23.5 (3.5)	0.377
Current smokers, %	14.4	12.7	<0.001
Time spent walking <0.5 h/d, %	40.3	40.3	0.055
Depression,[b] %	36.3	33.7	0.001
History of disease, %			
Hypertension	39.4	43.4	<0.001
Diabetes mellitus	11.4	12.2	0.120
Stroke	3.7	4.8	<0.001
Myocardial infarction	4.5	5.5	0.002
Cancer	6.2	8.8	<0.001

LTCI, long-term care insurance; SD, standard deviation.

[a]Obtained by using X^2 test for variables of proportion and one-factor analysis of variance for continuous variables (missing value exclude).

[b]Depression and Suicide Screen score ≥2.
